# Recent Progress in Synthesis, POM Analyses and SAR of Coumarin-Hybrids as Potential Anti-HIV Agents—A Mini Review

**DOI:** 10.3390/ph16111538

**Published:** 2023-10-31

**Authors:** Mustapha Suleiman, Faisal A. Almalki, Taibi Ben Hadda, Sarkar M. A. Kawsar, Subhash Chander, Sankaranarayanan Murugesan, Ajmal R. Bhat, Andrey Bogoyavlenskiy, Joazaizulfazli Jamalis

**Affiliations:** 1 Department of Chemistry, Faculty of Science, Universiti Teknologi Malaysia, Johor Bahru 81310, Johor, Malaysia; masge007@gmail.com; 2 Department of Chemistry, Sokoto State University, Birnin Kebbi Road, Sokoto 852101, Nigeria; 3 Department of Pharmaceutical Chemistry, Faculty of Pharmacy, Umm Al-Qura University, Mecca 21955, Saudi Arabia; malkifaisal2@gmail.com (F.A.A.); taibi.ben.hadda@gmail.com (T.B.H.); 4 Laboratory of Applied Chemistry & Environment, Faculty of Sciences, Mohammed Premier University, MB 524, Oujda 60000, Morocco; 5 Laboratory of Carbohydrate and Nucleoside Chemistry, Department of Chemistry, University of Chittagong, Chittagong 4331, Bangladesh; akawsar@cu.ac.bd; 6 Amity Institute of Phytochemistry & Phytomedicine, Amity University Uttar Pradesh, Noida 201313, India; subhashsaininiper@gmail.com; 7 Medicinal Chemistry Research Laboratory, Birla Institute of Technology & Science Pilani (BITS Pilani), Pilani Campus, Pilani 333031, India; murugesan@pilani.bits-pilani.ac.in; 8 Department of Chemistry, R.T.M. Nagpur University, Nagpur 440033, India; bhatajmal@gmail.com; 9 Research and Production Center for Microbiology and Virology, Almaty 050010, Kazakhstan

**Keywords:** coumarin, HIV, AIDS, reverse transcriptase, HAART, POM, synthesis

## Abstract

The human immunodeficiency virus (HIV) is the primary cause of acquired immune deficiency syndrome (AIDS), one of the deadliest pandemic diseases. Various mechanisms and procedures have been pursued to synthesise several anti-HIV agents, but due to the severe side effects and multidrug resistance spawning from the treatment of HIV/AIDS using highly active retroviral therapy (HAART), it has become imperative to design and synthesise novel anti-HIV agents. Literature has shown that natural sources, particularly the plant kingdom, can release important metabolites that have several biological, mechanistic and structural representations similar to chemically synthesised compounds. Certainly, compounds from natural and ethnomedicinal sources have proven to be effective in the management of HIV/AIDS with low toxicity, fewer side effects and affordability. From plants, fungi and bacteria, coumarin can be obtained, which is a secondary metabolite and is well known for its actions in different stages of the HIV replication cycle: protease, integrase and reverse transcriptase (RT) inhibition, cell membrane fusion and viral host attachment. These, among other reasons, are why coumarin moieties will be the basis of a good building block for the development of potent anti-HIV agents. This review aims to outline the synthetic pathways, structure–activity relationship (SAR) and POM analyses of coumarin hybrids with anti-HIV activity, detailing articles published between 2000 and 2023.

## 1. Introduction

For over thirty years, human immunodeficiency virus type 1 (HIV-1) infection has been a major global health problem, affecting more than 37 million people worldwide [1]. Acquired immunodeficiency syndrome (AIDS), which is a result of the human immunodeficiency virus (HIV), provokes fatal infections and numerous related diseases. The human immunodeficiency virus is characterised by the destruction of the immune system, which leads to improper functioning of the human immune system, the latter being characterised by the ability to fight any incoming infection and resulting in the well-being of the body [2]. HIV is a lentivirus, otherwise known as a “slow” virus, which ultimately causes acquired immunodeficiency syndrome (HIV-1 and HIV-2). This is commonly known as acquired immunodeficiency syndrome (AIDS) [1,3]. According to the World Health Organisation (WHO), approximately 38 million people worldwide have been infected with HIV, and about 2 million new cases are diagnosed each year [4].

One of the well-known methods, Petra/Osiris/Molinspiration (POM) analysis, has been used frequently to create two-dimensional models to discover and suggest the kind of pharmacophore site that influences the biological activity with a change in the chemical. POM can predict molecule biological activities and depict the relationships between steric/electrostatic properties and biological activity as pharmacophore sites [5]. 

## 2. Bibliometric Analysis

There are only a few linked publications available in the WOS database about the keywords we used for the search. Keywords such as “coumarin”, “coumarin-hybrids”, “HIV”, and “SAR” were used to search for the topic search criteria on 3 September 2023, and 1302 research items were discovered from 2000 to 2023. 

Recently, the development of various anti-HIV therapies has changed AIDS infection from a deadly disease to a more manageable yet chronic ailment. Such agents include nucleoside and nucleotide reverse transcriptase inhibitors, non-nucleoside reverse transcriptase inhibitors, protease inhibitors, fusion inhibitors, and co-receptor inhibitors. The treatment, however, of HIV infection has experienced many challenges, ranging from the severe side effects experienced to the mutation of the virus; these two have been lingering problems in the therapy of the disease, making the development of novel anti-HIV drugs necessary [6]. In response to the issue, scientists have developed novel anti-HIV agents that can effectively address the resistance and consequent toxicity problems reported by some anti-HIV agents currently used to treat HIV/AIDS. Due to this, “highly active antiretroviral therapy,” also known by the acronym (HAART), has become a new treatment [5,7], which has yielded a positive response from patients as reported by [8,9]. Antiretroviral therapy (ART) or combined antiretroviral therapy (cART) are other terms for this treatment regimen. The co-administration of different drugs that inhibit viral replication by distinct methods, so that the propagation of a virus resistant to a single agent is stopped by the action of the other agents in the combined regimen, is a major component of HAART. This therapy, HAART” is a type of medication that involves the simultaneous usage and administration of both non-nucleoside HIV-1 RT inhibitors and some protease inhibitors to increase the drug’s efficacy as reported by Olomola et al. [10]. Reports have shown that typical tenofovir–emtricitabine combination (nucleoside reverse transcriptase inhibitors) in combination with integrase strand transfer inhibitors or non-nucleoside reverse transcriptase inhibitors is used as a standard treatment for treatment-naive patients [11,12,13]. Another and most important goal of the HAART regimen is to reduce HIV transmission to others. In certain studies, the HAART regimen has been shown to lower the risk of sexual transmission to partners to practically zero [14]. Mother-to-child transmission during breastfeeding and pregnancy has also been drastically reduced [15,16,17]. Although highly active antiretroviral therapy (HAART) enables long-term control of virus replication in many individuals, it is not without unintended side effects, some of which are already emerging in older populations receiving long-term treatment [18]. 

**Figure 1:**
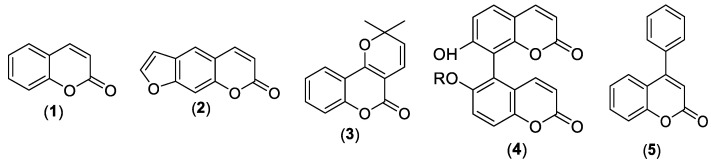
Structure of different coumarins.

Natural products have been used to treat viral illnesses for thousands of years and are increasingly important in medication research and development [19]. The exploration of medicinal plants and natural products that might yield affordable and effective therapeutic agents in the management of HIV/AIDS is a response to the declaration by the World Health Organisation (WHO) in 1989 [20,21]. Natural coumarins (Figure 1) are classified into several types depending on their chemical diversity and complexity: simple coumarins (**1**), furanocoumarins (**2**), pyranocoumarins (**3**), biscoumarins (**4**), and additional coumarins such as phenylcoumarins (**5**) [22,23,24]. Coumarins **1** (2*H*-chromen-2-one or 2*H*-1-benzopyran-2-one), secondary metabolites that belong to the family of benzopyrones, are found in a variety of plant parts, such as roots, seeds, nuts, flowers, and fruit [25,26].

It has been established that compounds possessing a coumarin moiety have exhibited a variety of intriguing and exciting activities due to their varying pharmacological properties, such as anti-bacterial activity [4,24,25], anti-cancer [4,25,26], anti-coagulant [4,27], anti-oxidant [28], anti-fungal [4,29,30,31], anti-tubercular [4,32,33] and most importantly for this research, anti-viral and anti-HIV activity [4,34,35]. 

Coumarin possesses the ability to perform several important interactions with diverse proteins, enzymes and receptors, ranging from hydrogen bonding, Van der Waals forces, chelation activities, hydrophobic interaction, and many more, which makes coumarin and its derivatives have a vast application as inhibitors of HIV protease: an integrase (IN) inhibitor, an inhibitor of viral DNA replication, a viral protein regulator (VPR) and also as an inhibitor of reverse transcriptase (RT). The ability to exert and manifest multiple anti-HIV activities as well as having the potential to overcome persistent resistance is a prominent property of coumarin and its derivatives [4,34]. Hybridization of molecules involves the combination of more than one pharmacophore to produce one hybrid compound that could inhibit HIV by single or multiple mechanisms and counterbalance the side effects that manifest because of the usage of a particular compound, or different hybrids that provide a novel anti-HIV activity with a much better result [4,36].

Many studies have shown that coumarin and the analogues of coumarin have exhibited a range of antiviral characteristics and more specifically act as HIV-1 inhibitors [2,4,5,34,35]. To the best of our knowledge, there has never been a comprehensive review that has detailed the coumarin hybrids with anti-HIV potential. This review aims to provide a comprehensive analysis of coumarin hybrids, focusing on their synthesis, biological activities, and structure–activity relationship (SAR). Specifically, it will explore their potential as anti-HIV agents, with a particular emphasis on articles published between 2000 and 2023. 

## 3. Coumarin Hybrids

In 2004, Lan Xie and colleagues [36] conducted a study in which they synthesised hydroxymethyl (3′*R*,4′*R*)-3′,4′-di-O-(*S*)-camphanoyl-(+)-cis-khellactone (DCK) derivatives. The aim was to enhance the oral bioavailability and water solubility of dicamphanoyl khelactone analogues [37] while evaluating their potential to inhibit HIV-1 replication in CD4 cells. These DCK derivatives belong to the category of pyranocoumarins, which are considered anti-HIV inhibitors and can be classified into four distinct structural classes.

**Scheme 1:**
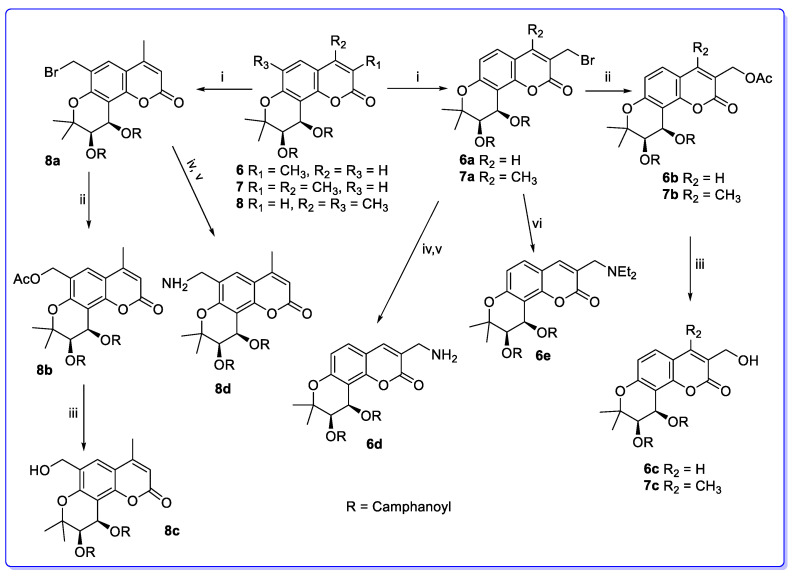
Synthetic pathways for the synthesis of hydroxymethyl DCK analogues. Conditions and reagents: (**i**) *N*-bromosuccinimide in benzene, reflux; (**ii**) NaOAc, acetic anhydride, reflux; (**iii**) reflux, HCl (2N) in EtOH; (**iv**) diethylamine in toluene, reflux; (**v**) hexanemethylenetetramine in CHCl_3_, reflux; (**vi**) EtOH, HCl (2N), 100 °C.

The synthesis process for the analogues began with the preparation of methylated DCKs from methylated 7-hydroxycoumarin, as reported [38,39]. The methylated DCKs (**6**, **7** and **8**) were treated with *N*-bromosuccinimide to yield 3-bromomethyl (**6a** and **7a**) and 6-bromomethyl (**8a**) substituted DCKs with a product yield range of 67–77%. Depending on the quantity of bromosuccinimide used, small amounts of 3- or 6-dibromoethyl compounds were also formed. By employing sodium acetate, the bromomethyl-DCK derivatives (**6a**, **7a** and **8a**) were reacted with acetic anhydride to generate the corresponding acetoxymethyl-DCK derivatives (**6b**, **7b** and **8b**) with 79–84% as the product yield. Subsequent acidic hydrolysis of these compounds produced the corresponding hydroxymethyl-DCK derivatives (**6c**, **7c** and **8c**) and the yield was found to be above 85%. Further treatment of **6a** and **8a** with hexanemethylenetetramine, followed by hydrolysis, led to the formation of aminomethyl DCK (**6d** and **8d**). Additionally, the treatment of compound **6a** with diethylamine resulted in the production of 3-dimethylaminomethyl DCK (**6e**) with 71% yield of product [36], as illustrated in Scheme 1.

The anti-HIV activity of all the newly synthesised analogues was tested using 4-methyl-DCK, DCK and AZT (zidovudine/azidothymidine) as the reference drugs. Based on the biological studies, the EC_50_ values of compounds **7c** and **6c** (0.004 and 0.029 µM) were the most active compounds when compared to AZT and DCK standards. Compounds **6b**, **7b** and **6a** showed EC_50_ values better than those of AZT but similar to the values of DCK. The excellent potency exhibited by compound **7a** with 0.00011 µM, as the EC_50_ value is worth noting, was more potent than the 4-methyl-DCK standard. Moderate anti-viral activity was observed in the compounds **8a**, **8c** and **8d** but even then, they exhibited better potency than AZT, although not as potent as DCK and the 4-methyl-DCK standards. 

**Figure 2:**
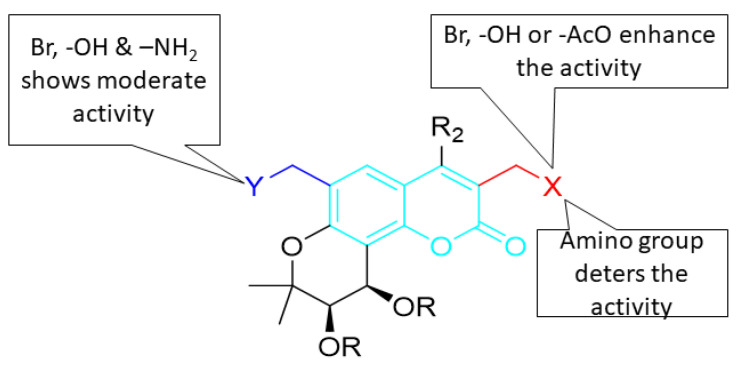
SAR representation of hydroxymethyl DCK analogues.

The structure–activity relationship (SAR) of the synthesised compounds indicates that bromomethyl, hydroxymethyl, or acetoxymethyl groups at the 3-position show better or similar activity than seen in compound **7a**. A negative effect was seen in compounds that possess amino moieties, as seen in compounds **6d** and **6e**, which is an indication that the anti-HIV activity is not favoured by a diethylaminomethyl or aminomethyl group at the 3-position. The SAR can be generalised by saying favourability in anti-HIV activity is better in the 3-position of DCK but not the 6-position (Figure 2).

**Scheme 2:**
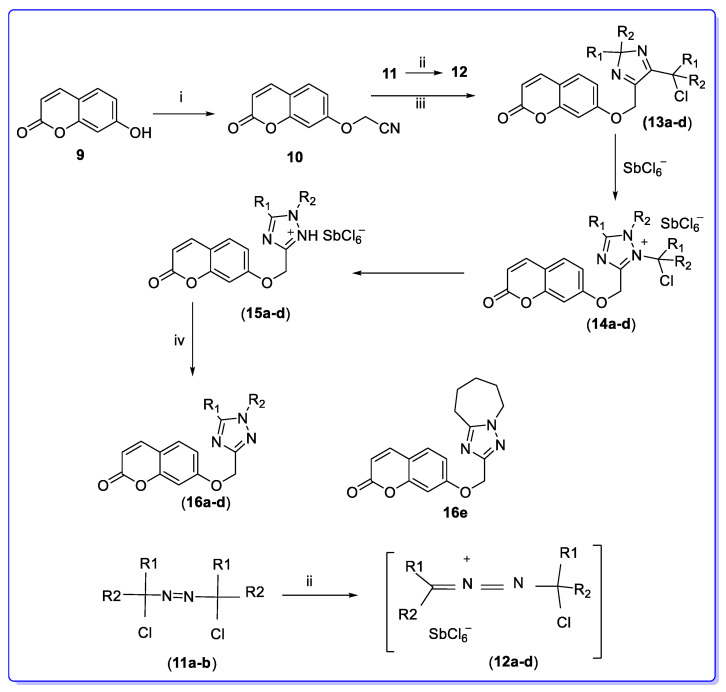
Synthetic route for the synthesis of 1,2,4-triazolylcoumarin hybrid. Reagents and conditions: (**i**) ClCH_2_CN; (**ii**) CH_2_Cl_2_, SbCl_5_, −60 °C; (**iii**) −60 °C to 23 °C, CH_2_Cl_2_; (**iv**) NH_3_, NaHCO_3_, MeCN, 2 h, 0 °C. **11a**–**16a** R_1_ = R_2_ = CH_3_; **b** R_1_ = R_2_ = Et; **c** R_1_ = R_2_ = Et; **d** R_1_ = CH_3_, R_2_ = *i-*Pr, **e** R_1_ = R_2_ = (CH_2_)_5_.

Al-Soud et al. [39] synthesised a new hybrid of 1H-1,2,4-triazolylcoumarin due to the enormous biological activities exhibited by 1,2,4-triazole [40] and coumarin [41]. The synthetic pathway for the synthesis of triazolylcoumarin hybrids is shown in Scheme 2, starting with 7-hydroxycoumarin **9**. 2-Chloroacetontrile was treated with 7-hydroxycoumarin **9** to prepare 2-(2*H*-benzopyran-7-yloxy)acetonitrile **10** with 82% as the reaction yield, which was used as the precursor to synthesise 1,2,4-triazole in DMF containing K_2_CO_3_. On treatment with SbCl_5_ at −60 °C, intermediate **12** was afforded from α,α′-dichloroazo compounds **11**. The colour of the mixture changes from orange to brown at around −30 °C, an indication that a cycloaddition reaction has occurred between nitrile **10** and cumulenes **12a**–**e** to generate inseparable 1,2,4-triazolium hexachloroantimonates **13a**–**e**. A spontaneous rearrangement occurred from compounds **13a**–**e** to the intermediate **14a**–**e** with an increase in temperature above −30 °C. The coumarin salts **15a**–**e** were produced from **14a-e** via 1,2-migration and the subsequent elimination of the CMe_2_Cl group. In the presence of NaHCO_3_ and aqueous NH_3_, an in situ deprotonation of coumarin salts **15a**–**e** afforded the desired compound **16a**–**e** with a percentage yield of 72–85% [39].

**Scheme 3:**
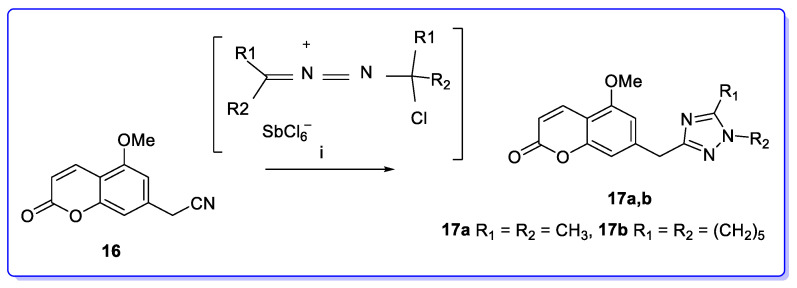
Synthetic pathway for the synthesis of novel hybrid **17.** Reagents and conditions: (**i**) NH_3_, NaHCO_3_, MeCN, 2 h, 0 °C.

To further explore the potentiality of coumarin with 1,2,4 triazole, the scientists also synthesised another analogue following the same route as depicted in their previous work shown in Scheme 2 above. However, in this case, the research was extended on coumarin derivatives with 1,2,4-triazoles by adding a cyanomethyl group in position 7 of the backbone, so the resulting starting material was now (2-(5-methoxy-4-methyl-2*H*-benzopyran-7-yloxy) acetonitrile) **16**. Scheme 3 illustrates the cycloaddition reaction that occurs between cumulenes **12a**–**e** and compound 2-(5-methoxy-4-methyl-2*H*-benzopyran-7-yloxy) acetonitrile. Compounds **17a** and **b** were synthesised in 85 and 75% yield. 

**Figure 3:**
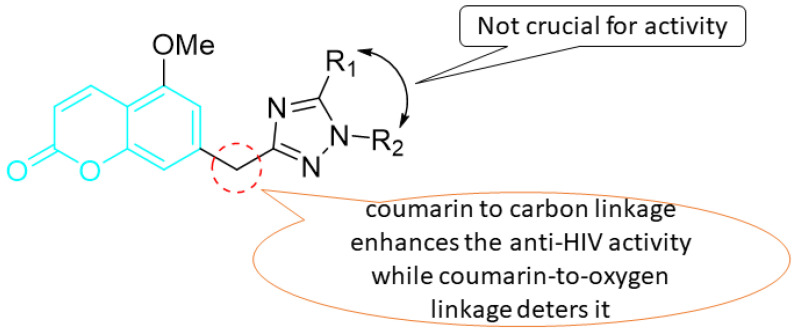
SAR representation of 1H-1,2,4-triazolycoumarin hybrids.

The anti-HIV activity of the newly synthesised compounds was carried out on compounds **10**, **16a**–**e** and **17b** using the HIV-2 ROD strain and the III_B_ strain for HIV-1. The inhibition was monitored in MT-4 cells by virus-induced cytopathy (in vitro). A very weak inhibitory activity was noticed in all the tested compounds. Both HIV-2 and HIV-1 replication were not inhibited by the compounds. A promising activity was noticed in compound **17b** with IC_50_ value >0.17 µM, selectivity index of <1.0 and CC_50_ of 0.17 µg/mol. The suggestion obtained from the SAR studies is that a higher anti-HIV activity was exteriorized by coumarin hybrids possessing carbon–coumarin linkage than the corresponding series of hybrids possessing oxygen linkage (Figure 3). 

**Scheme 4:**
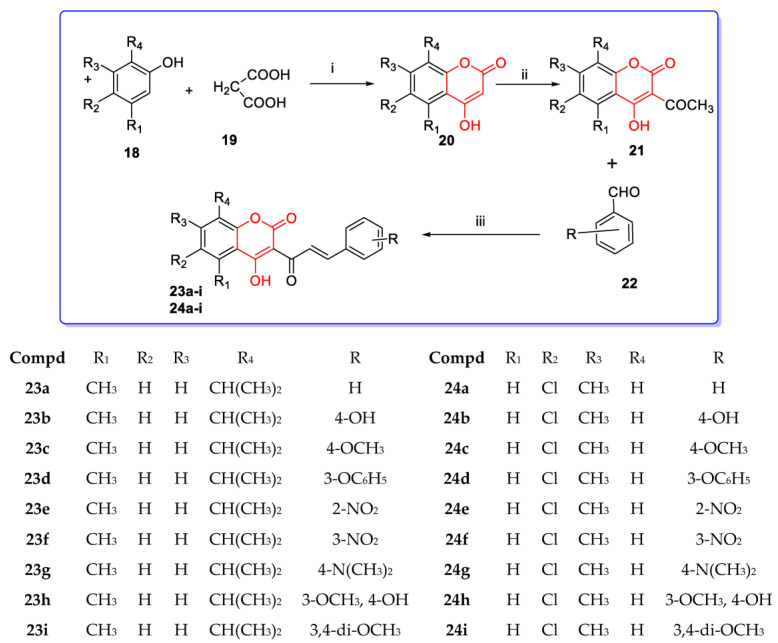
A synthesis pathway for the synthesis of **23a**–**i** and **24a**–**i**. Reaction and condition; (**i**) POCl_3_, anhydrous ZnCl_2_, 70 °C; (**ii**) POCl_3_, glacial acetic acid; (**iii**) piperidine, CHCl_3_, 80 °C.

Still on the hunt for a novel and potent anti-HIV drug, Trivedi et al. synthesised new coumarinyl chalcone analogues from two closely structurally related coumarins: 4-hydroxy-6-chloro-7-methylcoumarin and 4-hydroxy-8-isopropyl-5-methylcoumarin. The duo were their respective chalcones. The synthetic procedure is depicted in Scheme 4. The starting materials used for this synthesis are malonic acid **19** and phenol **18**. In the presence of anhydrous ZnCl_2_, Lewis acid and phosphorus oxychloride (POCl_3_), substituted coumarins **20** are prepared by the condensation of appropriately substituted malonic acid **19** and phenol **18**. The resulting substituted coumarin is acetylated using POCl_3_ and glacial acetic acid as acetylating agents. The reaction proceeds to completion with the reaction with piperidine and CHCl_3_ to produce the target compounds **23a**–**i** and **24a**–**i** with a percentage yield range of 69–79% [42]. 

**Figure 4:**
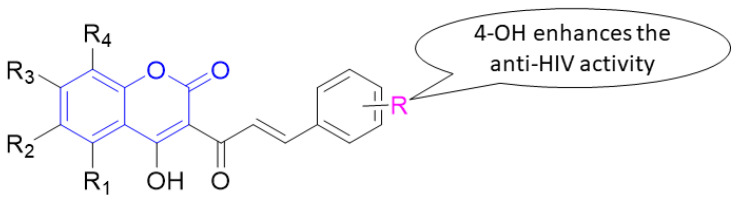
SAR presentation of new coumarinyl chalcone analogues **23a**–**i** and **24a**–**i.**

Using MT-4 cells, the antiviral activity screening of all the synthesised compounds was carried out against HIV-1 (IIIB) and HIV-2 (ROD) using zidovudine (AZT) as the reference drug for comparison purposes. Unfortunately, all the synthesised compounds exhibited no anti-HIV activity against all the viruses evaluated. Each activity showed a cut-off concentration of ≥5-fold lower than the cytotoxic concentration. The structure–activity relationship is presented in Figure 4.

**Scheme 5:**
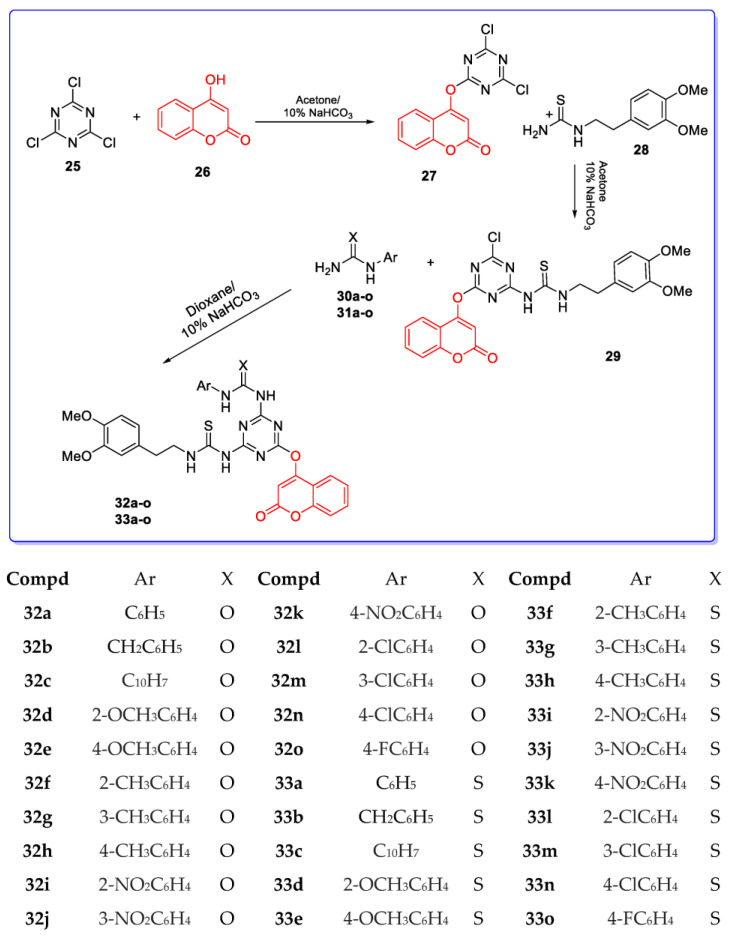
A synthetic pathway for the synthesis of PETT analogues.

As a result of the mass interest garnered recently by thiourea as an interesting moiety for the design of novel and potent anti-HIV compounds, the hybridization of s-triazine derivatives, coumarin derivatives, and phenyl ethyl amine derivatives has been performed by Patel et al. to synthesise novel PETT (phenyl ethyl thiazolyl thiourea) analogues as possible novel HIV inhibitors. The synthesis of the novel PETT analogues (Scheme 5) was initiated by the condensation of 2,4,6-trichloro-1,3,5-s-triazine **25** with 4-hydroxycoumarin **26** in the presence of acetone and 10% NaHCO_3_ to yield compound **27** as an intermediate. The intermediate formed is then further condensed with 3,4-dimethoxy phenyl ethyl thiourea **28** to afford an important intermediate **29**, which further undergoes substitution with various substituted phenyl thiourea/ureas (**30a**–**o/31a**–**o**) to produce the derivatives (**32a**–**o**) and (**33a**–**o**) of 3,4-dimethoxy phenyl ethyl-1,3,5-triazinyl thiourea with yield ranges of 53–79% and 50–75%, respectively [43].

The MTT (4,5-dimethyl thiazol-2-yl)-2,5-diphenyl tetrazolium bromide) method for the screening of antiviral activity was used for the biological activity to determine the inhibition of replication of HIV. The anti-HIV screening of these derivatives shows no selectivity for HIV-1 and HIV-2. Compound **30h** shows an EC_50_ value of >124 µM/g mL which indicates poor activity. A moderate activity against both HIV-1 and HIV-2 was noticed in compound **31k** with a selectivity index of 3 for both HIV-1 and HIV-2. Compounds **31c**, **31h** and **31n** with S.I of 9,3 and 11, respectively, against HIV-1 were also observed. So generally, both analogues exert poor potency and selectivity. 

**Figure 5:**
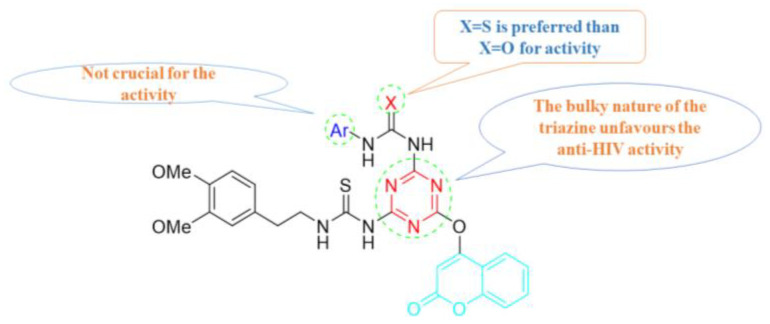
SAR representation of PETT analogues.

The structure–activity relationship (SAR) analysis (Figure 5) reveals that the poor activity of the synthesised analogue may be a result of the fact that the heterocyclic ring of the synthesised analogues contains a bulky triazine group. Overcrowding is also observed in the structure, which is a result of the ether linkage with the 4-hydroxycoumarin present, which disturbs the overall structure of the synthesised hybrids. 

In the year 2008, Reddy et al. [44], on the basis of the anti-HIV activity exhibited by Saquinavir **34** as the first inhibitor of protease to inhibit both HIV-2 and HIV-1 proteases and also the anti-HIV potency of (+)-calanolide A **35**, which is a pyranocoumarin derivative, prompted them to synthesise a new substituted *N^1^*-[1-benzyl-3-(3-*ter*t-butylcarbamoyl-octahydroisoquinolin-2yl)-2-hydroxypropyl]-2-[(2-oxo-2*H*-chromene-3-carbonyl)amino] succinimide as a novel anti-HIV agent. 


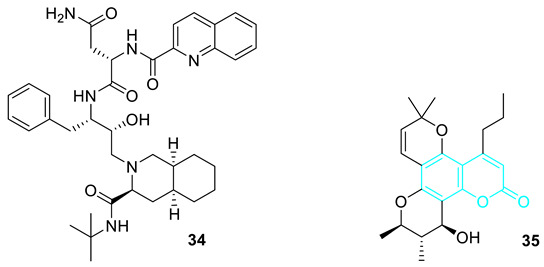


**Scheme 6:**
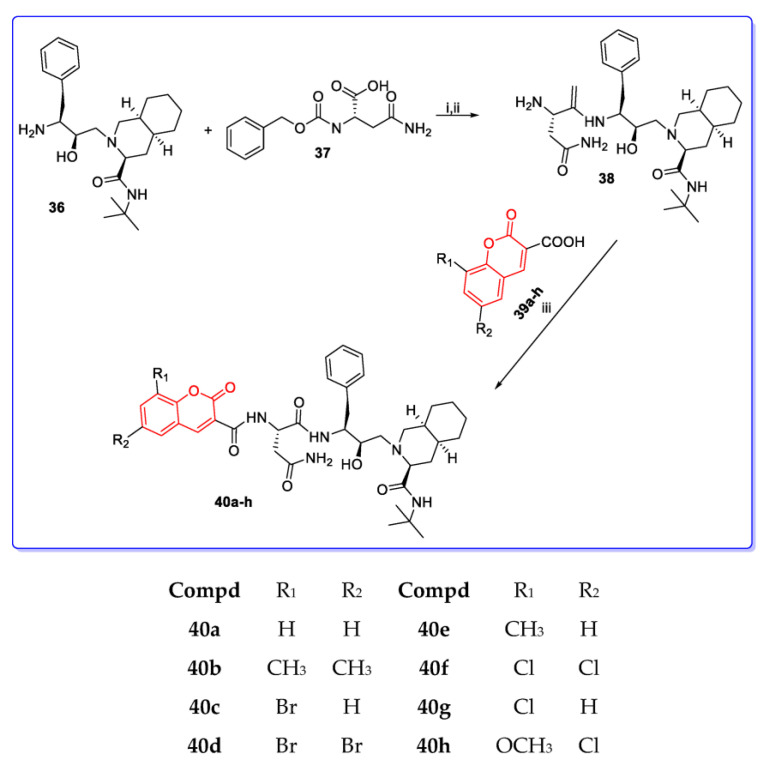
Synthetic pathway for the synthesis of hybrid **40a**–**h.** Reagents and conditions: (**i**) THF, DCC, TEA, 24 h, 20–25 °C, N_2_; (**ii**) EtOAc, H_2_, 5% Pd/C, 20–25 °C, 48 h; (**iii**) THF, DCC, TEA, 20–25 °C, 24 h, N_2_.

The synthesis (Scheme 6) began with the condensation of 2-(3-amino-2-hydroxy-4-phenyl-butyl) decahydroisoquinoline-3-carboxylic acid *tert*-butylamide **36** with *N*-benzyloxy carbonyl-*L*-asparagine **37** using triethylamine in dry hydrofuran for 24 h at room temperature and dicyclohexylcarbodiimide (DCC). The reaction was then deprotected with 5% Pd-C and hydrogen to prepare 2-amino-*N^1^*-[l-benzyl-3-(l-*tert*-butylcarbamoyloctahydroisoquinolin-2-yl)2-hydroxypropyl] succinamide **38**. Using the appropriate coumarin-3-carboxylic acid (**39a**–**h**), compound **38** was then condensed in the presence of triethylamine for 24 h at room temperature in dry hydrofuran and dicyclohexylcarbodiimide (DCC) to afford the product *N^1^*-[1-benzyl-3-(3-*ter*t-butylcarbamoyl-octahydroisoquinolin-2yl)-2-hydroxy- propyl]-2-[(2-oxo-2*H*-chromene-3-carbonyl)amino] succinimide **40.**

MT-4 cells were used to monitor the inhibition of the induced cytopathic effect, and the activity was estimated by the MTT method. Using azidothymidine (AZT) as the reference drug, the synthesised compounds **40a**–**h** were tested for anti-HIV activity against HIV-1 and HIV-2. Compound **40g** exhibited greater activity against both HIV-1 and HIV-2 infections, but compound **40c** showcased poor anti-HIV activity when tested against both HIV-1 and HIV-2. Moderate activity against both HIV-1 and HIV-2 was manifested by compounds **40d** and **40f**, while compounds **40e** and **40h** showed no activity against HIV-1 and HIV-2.

In summary, the structure–activity relationship (SAR) for compounds **40b**, **40d**, **40f**, and **40g** demonstrate that appropriate substitutions at positions 6 and 8 of the coumarin ring are necessary for the anti-HIV activity of the synthesized compounds.

**Figure 6:**
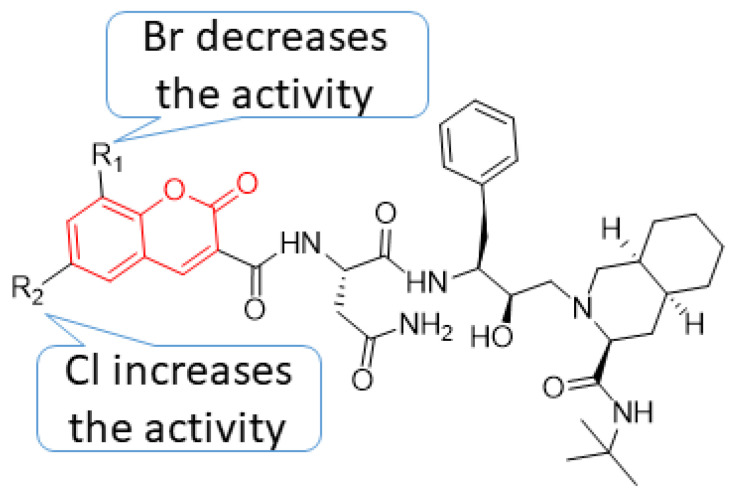
SAR representation of hybrids **40a**–**h.**

Compound **40g** shows that the chlorine atom at position 8 of the coumarin ring enhanced the anti-HIV activity of the hybrids (Figure 6).

In the year 2008, Al-Soud and co-workers [45] synthesised coumarin hybrids on the basis of the anti-HIV activity exhibited by coumarin derivatives and also the anti-HIV activity displayed by capravirine **41** with an EC_50_ value of 0.0014 µg mL^−1^, CC_50_ value of 11 µg mL^−1^ and SI (selectivity index) value of 7857, which is a compound containing an imidazole moiety [45,46,47]. The standard drug used for comparison in this research is efavirenz which has an EC_50_ of 0.003 and CC_50_ of 40 µg mL^−1^ respectively.


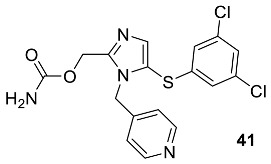


**Scheme 7:**
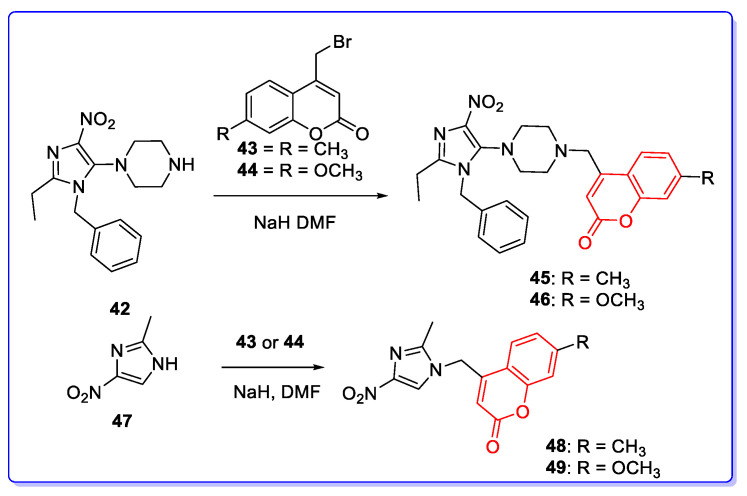
A synthetic pathway for the synthesis of **45**, **46**, **48** and **49**.

The starting material for the synthesis was compound **42**. The two 4-bromomethylcoumarins **43** and **44** interacted with **42** in dimethyl formamide (DMF) to give the corresponding derivatives **45** and **46** with 43% and 46% yields, respectively. Following purification, Scheme 7 illustrates the synthesis of compounds **48** and **49** by treating 47 in DMF with **43** and **44**, with yields of 70 and 78%, respectively.

**Scheme 8:**
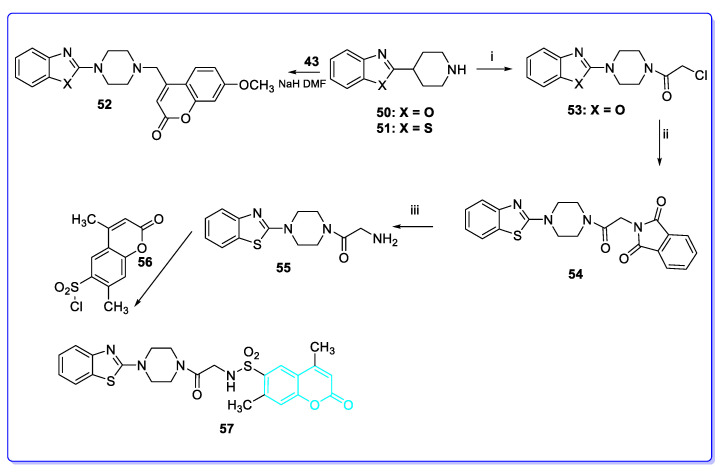
Synthesis of compound **57.** Reagents: (**i**) chloroacetyl chloride; (**ii**) K_2_CO_3_, potassium phthalimide; (**iii**) hydrazine hydrate.

Al-Soud et al. [45] further synthesised another coumarin derivative that contains benzothiazole and benzoxazole moieties (Scheme 8). The derivatives of benzoxazole **50** gave the compound **52** with 95% yield in the presence of NaH in DMF. 2-Chloroacetylchloride reacted with compound **51** to afford compound **53** (67% yield), which was treated with potassium phthalimide in the presence of K_2_CO_3_ to produce compound **54** (90% yield). Treatment of the resulting mixture with hydrazine hydrate converted it to the corresponding amine **55** (82% yield). Coumarin sulfonyl chloride **56** was used to sulfonate compound **55** in the presence of triethylamine to give the sulphonamide derivative **57** as the final product with 71% yield.

**Scheme 9:**
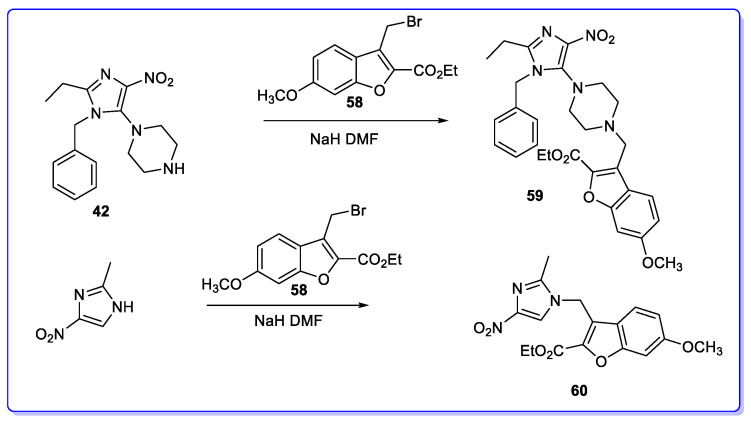
Synthesis of compounds **59** and **60**.

Still on the work of Al-Soud, Scheme 9 depicts the synthesis of imidazole analogues **59** and **60**. In the presence of benzofuran, the imidazole derivatives **59** and **60** with a yield of 63 and 48%, respectively were synthesised by the treatment of **42** and **47** with **58** in the presence of NaH in DMF.

Using in vitro anti-HIV assay, compounds **45**, **46**, **48**, **49**, **59** and **60** were screened against both HIV-1 (strain III_B_) and HIV-2 (strain ROD) in MT-4 (human *T-*lymphocyte) cells. The result shows no inhibition at CC_50_ higher than EC_50_ with the standard anti-HIV agents capravirine and efavirenz (EFV). However excellent activity when compared to other compounds was displayed by compounds **48** and **49**. HIV-1 inhibition was shown by compound **48** with an EC_50_ value of 1.22 µg/mL and an EC_50_ value of 0.51 µg/mL for HIV-2 inhibition. For compound **49**, HIV-1 inhibition was noted as possessing an EC_50_ value of 1.45 µg/mL and HIV-2 inhibition with an EC_50_ value of 1.78 µg/mL [45]. 

Based on the anti-HIV activity exhibited by s-triazine (probably as a result of the possession of the characteristics of Het–NH–Ph–U motif derivatives [48], 2-coumarin-4-yloxy-4,6-substituted-s-triazine derivatives were synthesised and their in vitro anti-HIV activities were tested, as reported by [49].

**Scheme 10:**
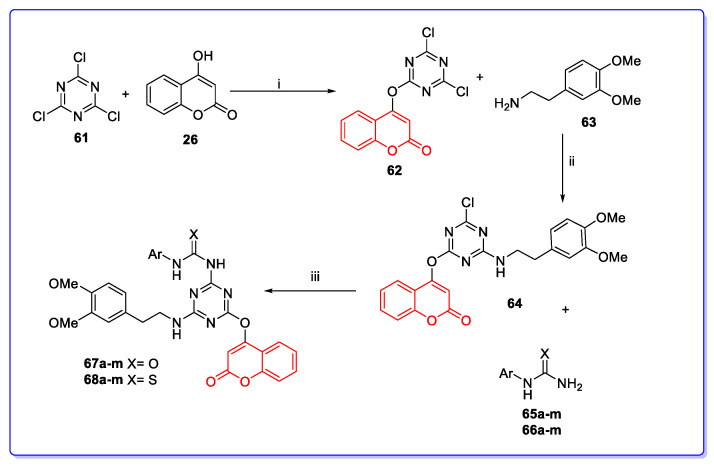
Synthetic pathway for hybrids **67a**–**m** and **68a**–**m**. Reagents and conditions: (**i**) 10% NaHCO_3_/acetone (**ii**) acetone/10% NaHCO_3_ (**iii**) 10% NaHCO_3_/dioxane.

As shown in Scheme 10, 2,4,6-trichloro-1,3,5-s-triazine **61** was condensed with 4-hydroxycoumarin **26** to afford an intermediate **62** (85% yield). In the presence of NaHCO_3_, the intermediate **62** reacted with 3,4-dimethoxyphenylethylamine **63** to form compound **64** (72% yield). To prepare the novel 3,4-dimethoxyphenylethyl-1,3,5-triazinyl amine derivatives **67a**–**m** with a percentage yield range of 56–74% and **68a**–**m** with a percentage yield of 55–75%, variously substituted phenyl urea/thiourea compounds **65a**–**m** and **66a**–**m** were reacted with intermediate **64** in the presence of NaHCO_3_ in dioxane. 

**Scheme 11:**
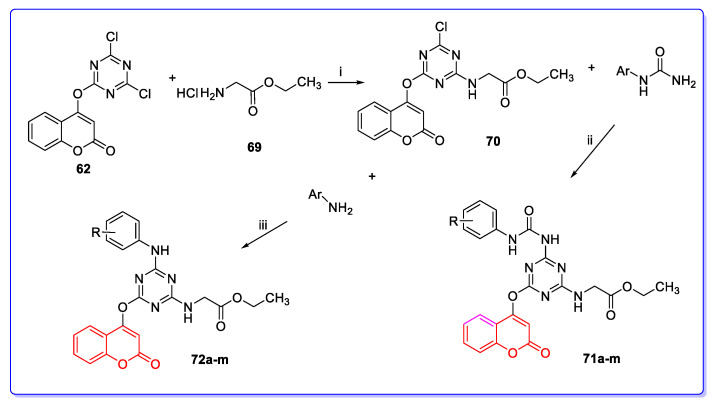
Synthesis of compounds **71a**–**m** and **72a**–**m**. Reagents and conditions: (**i**) 10% NaHCO_3_/acetone (**ii**) acetone/10% NaHCO_3_ (**iii**) 10% NaHCO_3_/dioxane.

Another synthetic pathway (Scheme 11) showed the replacement of two chlorine atoms on intermediate **62**, where intermediate **62** was reacted with glycine ethyl ester hydrochloride **69** to afford an important intermediate **70** (60% yield). On further treatment of **70** with various aryl ureas, new compounds **71a**–**m** were formed with the yield range of 54–69%, and several aryl amines afforded **72a**–**m** with a yield range of 56–67%.

To determine the ability of the synthesised compounds to inhibit the replication of HIV-1 (IIIB) and HIV-2, MT-4 cells were used for the screening utilising the in vitro screening method. For the purpose of comparison, nevirapine was used as a standard reference drug. To further test the efficacy of the synthesised compounds, the compounds that proved to be potent were also tested against HIV-RT double mutant strains (K103N and Y181C) using efavirenz as the standard reference drug. Compounds **68d** and **68l** were found to be active against HIV-1 with IC_50_ values of 8.33 µg/mL and 10 µg/mL, respectively. For the modified analogues, compound **68d**, with an IC_50_ value of 1.07 µg/mL and SI of 4.3, was found to be the most potent of the synthesised compounds. A significant inhibition potential was noticed in compounds **66g** and **66j** with IC_50_ values of 6.86 µg/mL and 7.16 µg/mL, respectively, and a common SI of 7.

The structure–activity relationship (SAR) indicates that compound **68d**, with a methyl group at position 4 (4-CH_3_), and compound **68c**, which possesses an ethoxy group at position 4 (4-OEt) displayed selective inhibition of HIV-1. The SAR studies on the modified compounds (**65** and **66a**–**m**) also deduced that compounds **66d** and **66g**, which have para-substituted chloro and methyl groups, exhibited a selective inhibition of HIV-1. The highest potency against HIV-1 was observed in compound **66d** with a para-substituted methyl group (4-CH_3_). As part of the research programme embarked on by Olomola et al. [10] for the design, synthesis and evaluation of coumarin derivatives, a group of coumarin hybrids was designed that contain both triazolothymidine and coumarin moieties as potential dual-action RT and HIV-1 PR inhibitors. 

**Scheme 12:**
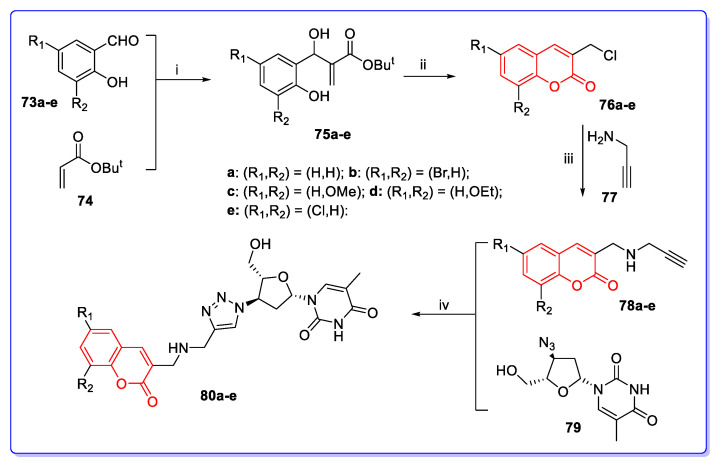
Synthesis of AZT-coumarin derivatives **80a**–**e**. Reagents and conditions (**i**) CHCl_3_, DABCO, rt; (**ii**) AcOH, HCl, reflux; (**iii**) THF, propargylamine; (**iv**) Cu_2_SO_4_.5H_2_O, C_6_H_7_NaO_6_ (sodium ascorbate), H_2_O-THF.

The synthesis started with the production of the corresponding adducts **75a**–**e** (Scheme 12), which were afforded by the DABCO (1,4-diazabicyclo[2.2.2]octane/triethylenediamine) catalysed Baylis–Hillman reaction of t-butyl acrylate **74** and the salicylaldehyde **73a**–**e**. Compounds **76a**–**e** 3-(chloromethyl)coumarins were formed from the cyclisation of **75a**–**e** with HCl-AcOH. Propargylamine **77** further reacted with **76a**–**e** in THF to give the corresponding **78a**–**e** in a yield range of 52–80%. In the presence of copper (II) sulphate and ascorbic acid, the cycloaddition of **78a**–**e** with azidothymidine **79** catalysed by Cu(I), resulted in the analogues **80a**–**e** as the final hybrid with the yield range of 64–75% [50].

To screen the anti-HIV activity of the synthesised compounds, commercially available HIV-1 RT and HIV-1 PR kits were utilised. Promising anti-HIV activity against HIV-1 PR was apparent on the synthesised analogues with an IC_50_ value range of 21–29 µM which is as much as two or three times higher than the IC_50_ value of ritonavir with an IC_50_ value of 9.85 µM. When compared with the IC_50_ value of AZT 73, compounds **80a** and **80b** show comparable inhibitory activity.

**Figure 7:**
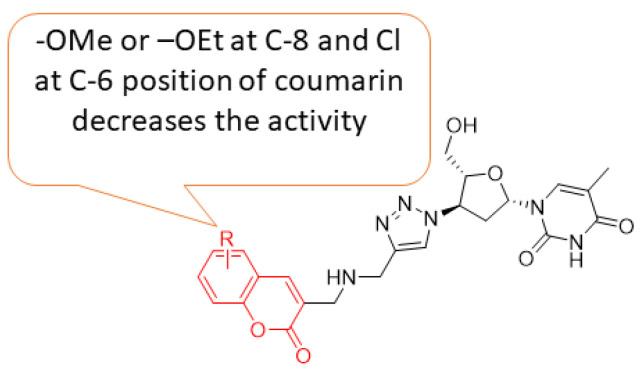
SAR representation of hybrids **80a**–**e**.

The structure–activity relationship (SAR) reveals that the unsubstituted analogue (**80a)** and the analogue (**80b**), which contain a bromine atom at the C-6 position of coumarin, are essential for the anti-HIV activity. However, a significant decrease in activity is observed in the compound that contains a chlorine atom at the C-6 position and an alkoxy group at the C-8 position of coumarin (Figure 7).

**Scheme 13:**
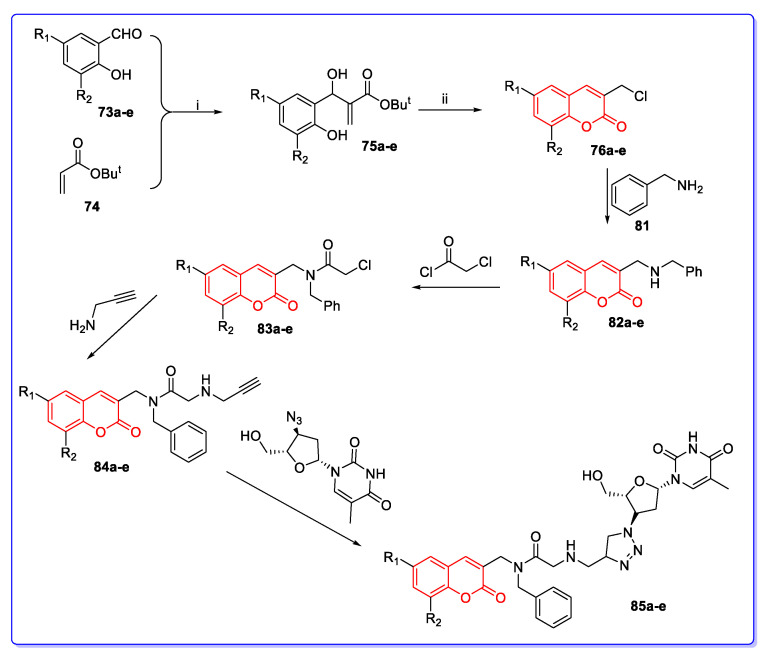
Synthetic pathways for the synthesis of coumarin–AZT hybrids **85a**–**e**. Reagents and condition: (**i**) CHCl_3_, DABCO, rt; (**ii**) AcOH, HCl, reflux.

Interestingly, in their quest to design and synthesise a potent compound with HIV-1 protease inhibition potential, Olomola et al. [10] further synthesised a new series of coumarin derivatives in the form of *N*-benzylated amido group derivatives **85a**–**e**. The synthesis of the modified analogues **85a**–**e** is illustrated in Scheme 13. The hybrids of **80a**–**e** and **85a**–**e** possess a common intermediate, **76a**–**e**. To form the secondary amine **82a**–**e**, the intermediate **76a**–**e** was reacted with benzylamine **81**. The corresponding chloroacetamide **83a**–**e** (78–98% yield) was afforded by the acylation of **82a**–**e** with chloroacetyl chloride, and subsequently, in THF, the resulting compound was reacted with propargylamine to give the alkynylated product **84a**–**e** (79–86% yield). Finally, with azidothymidine (AZT) **79**, compounds **84a**–**e** underwent cycloaddition in the presence of copper (II) sulphate and ascorbic acid to afford the hybrid **85a**–**e** with an isolated yield range of 70–80%.

**Figure 8:**
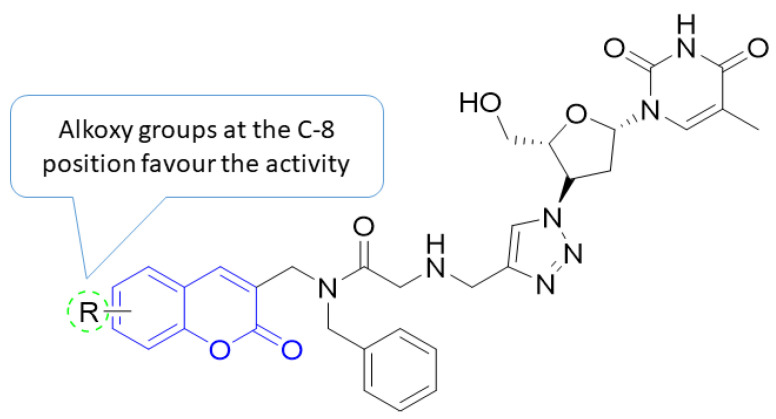
SAR representation of hybrids **85a**–**e**.

Under a variety of conditions, the hybrids **85a**–**e** were screened for HIV-1 RT and HIV-1 PR inhibition. Compounds **85a**–**e** showed an inhibition potential of up to 99% of HIV-1 RT. The compounds also showed HIV-1 PR inhibition, but not like that of the corresponding **80a**–**e**. They concluded that all the newly synthesised analogues **85a**–**e** exhibited a better IC_50_ value than azidothymidine **79**. The most active compounds against HIV-1 PR and HIV-1 RT were determined to be compounds **85a** and **85b**. From the anti-HIV result, the structure–activity relationship (SAR) revealed that the introduction of –OMe and –OEt (alkoxy groups) at the C-8 position of the coumarin will enhance the activity (Figure 8).

**Scheme 14:**
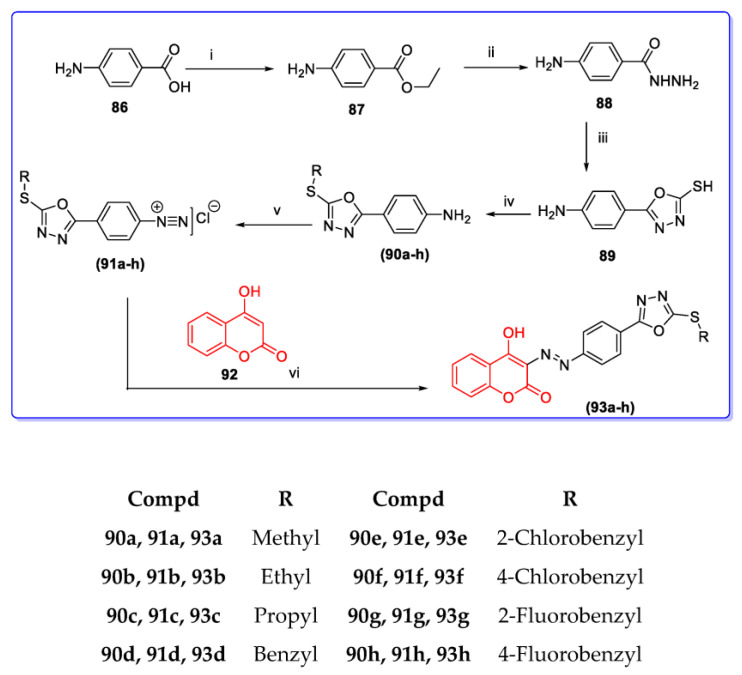
Synthetic pathways for the synthesis of diazocoumarin derivatives. Reagents and condition: (**i**) H_2_SO_4_, ethanol, reflux, 24 h (**ii**) NH_2_-NH_2_.OH, ethanol (6 eq.), reflux, 96 h (**iii**) CS_2_, ethanol, KOH, reflux, 4h (**iv**) 10% aq. NaOH, methanol, substituted aryl/alkyl halides, r.t (**v**) 10% sodium nitrite, 6 M HCl, 0–5 °C (**vi**) 10% aq. Na_2_CO_3_, stir, 0–5 °C.

In 2018, new diazocoumarin derivatives were designed and synthesised by Livani and co-workers to study the anti-HIV activity (integrase inhibition) of the synthesised analogues [51]. The anti-HIV activity evidenced in the bis-azo compound and coumarin scaffold was the reason for the hybridization, and so the 5-(halo-substituted) benzylthio-1,3,4-oxadiazole moiety and coumarin core were fused together to form the newly designed compound. Using ethanol and 4-aminobenzoic acid **86**, ethyl aminobenzoate **87** (74% yield) was first prepared via Fischer esterification using sulphuric acid as a catalyst. In absolute ethanol and excess hydrazine hydrate under reflux, ethyl 4-aminobenzoate **87** was converted to 4-aminobenzohydrazide **88** with a yield of 82%. In the presence of alcoholic potash and carbon disulfide, the mercaptho-1,3,4-oxadiazole ring **89** was closed on the carbohydrazide intermediate. In the presence of 10% NaOH as a base and methanol as a solvent, an *S*-alkylation or benzylation reaction was performed with alkyl or substituted benzyl halides to afford **90a**–**h** derivatives with a product yield range of 55–87%. In 10% sodium nitrite and 6M HCl, the diazonium salts of compound **91a**–**h** were prepared. Through the coupling reaction of 4-hydroxycoumarin **92** with the diazonium salts, the final derivatives **93a**–**h** were obtained in the range of 58–81% product yield (Scheme 14).

Via the single-cycle replication method, compounds **93a**–**h** were assayed for anti-HIV activity, which was measured as the inhibition rate (%) of HIV-1 in P24 expression in the Hela cell culture. At 100 µM concentration, all compounds **93a**–**h** showed anti-HIV activity in the range of 5–79%. Compound **93a**, with an inhibition rate of 79%, and compound **87f**, with cell viability of 52%, were superior to azidothymidine (AZT), with 58% viability. The results indicate that compound **87f** with 4-chlorobenzyl possesses the best anti-HIV activity.

**Figure 9:**
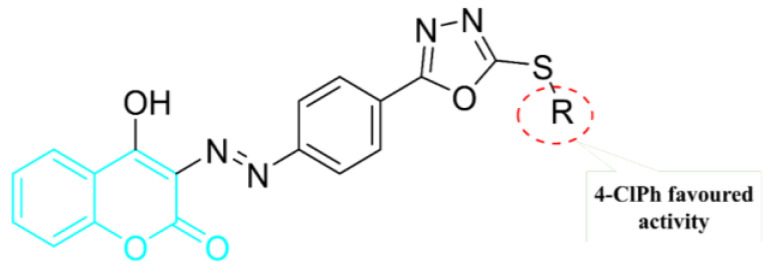
SAR representation of hybrids **93a**–**h**.

The structure–activity relationships (SAR) indicate that compound **87c** tolerates propyl substitution reasonably well, which shows similar inhibition to that of compound **93e**. Also, compound **93d** shows that introducing an unsubstituted benzyl group leads to a drastic reduction in activity. So, it was concluded that compound **93f** with a 4-chlorobenzyl group at C-5 shows the best anti-HIV activity (Figure 9).

Jesumoroti et al. [52] designed and synthesised a series of novel *N*′-(3-hydroxybenzoyl)-2-oxo-2*H*-chromene-3-carbohydrazide derivatives as potential HIV-1 integrase inhibitors by employing an approach called “scaffold-hopping”. The hybrids were produced by the combination of the coumarin moiety and hydrazide with the hope that 3-acyl-coumarin could mimic the diketo-acid moiety of raltegravir.

**Scheme 15:**
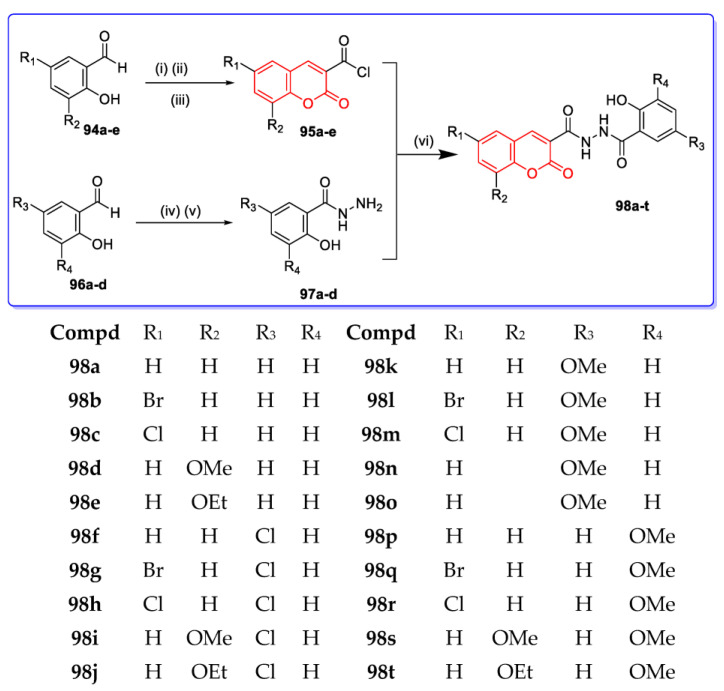
Synthetic pathways for synthesis of coumarin-3-carbohydrazide derivatives. Reagents and conditions: (**i**) piperidine, diethyl malonate, ethanol, CH_3_COOH, 80 °C, 12 h (**ii**) ethanol, NaOH, reflux (**iii**) oxalyl chloride, reflux, 8 h. (**iv**) CH_3_OH, Conc. H_2_SO_4_, reflux, 24 h (**v**) 65% N_2_H_4_.H_2_O, 80–90 °C, overnight. (**vi**) Na_2_CO_3_, room temperature, overnight.

Adapting the Knoevenagel condensation reaction process, ethyl 2-oxo-2*H*-chromene-3-carboxylate analogues **89a**–**e** were produced by synthesising the acid precursors **91a**–**e** from the condensation of salicylic aldehyde **88a**–**e** with diethyl malonate. Compounds **90a-e** were formed by the process of alkaline hydrolysis using NaOH. To afford the key intermediates, compounds **90a**–**e** were reacted with oxalyl chloride to form **91a**–**e**. Using H_2_SO_4_, esterification of substituted salicylic acid **92a**–**d** in methanol produced 2-hydroxybenzoate derivatives **93a**–**d** which on further hydrazinolysis afforded substituted 2-hydroxy benzohydrazides **94a**–**d**. Finally, in the presence of saturated Na_2_CO_3_, stirring **94a**–**d** with **91a**–**e** overnight produced **95a**–**t** with a variable yield range of 38–83% (attributed to the substitution pattern on the two rings (Scheme 15).

**Figure 10:**
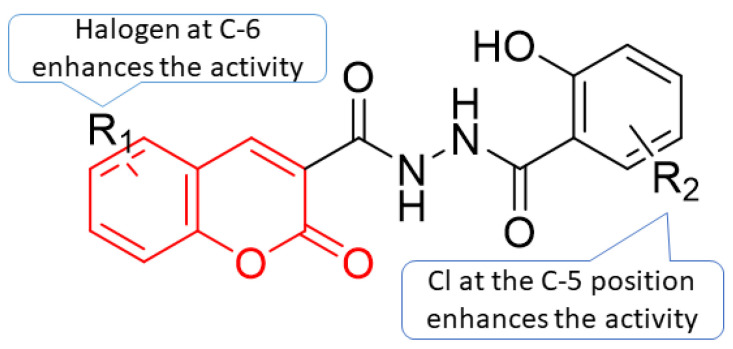
SAR representation of hybrids **98a**–**t**.

Chicoric acid was used as a standard in evaluating the HIV-1 IN inhibition of coumarin-3-carbohydrazide **98a**–**t** in the nanomolar range. The result of the HIV-1 IN activity showed that both hybrids **98a** and **98c** have an inhibition ability (IC_50_ = 14 nM) comparable to that of standard chicoric acid (IC_50_ = 10 nM). Structure–activity relationship (SAR) analysis revealed that the highest HIV-1 IN inhibiting activity was observed in hybrids that contain bromo- or chloro-substituents on ring A of the coumarin moiety while also having only the OH group on the salicyl moiety (ring C). The introduction of the methoxy group into the system decreased the HIV-1 IN activity. So generally, SAR revealed that the introduction of the chloro-group at the C-5 position (R_2_) of the phenyl group and the general introduction of the halogen atom in the C-6 position (R_1_) of the coumarin moiety enhanced the activity (Figure 10).

To achieve multiple inhibitions of virally coded enzymatic functions, coumarin-based scaffolds were exploited to synthesise sixteen novel 4-hydroxy-2*H*,5*H*-pyrano (3,2-*c*) chromene-2,5-dione hybrids [53]. The theoretical binding affinity of all the synthesised hybrids was calculated via modelling studies on both RT-associated ribonuclease H (RNase H) and HIV-1 IN sites. The compounds were later subjected to a biological assay to determine RNase H inhibitors and dual HIV-1 IN inhibitor hybrids.

**Scheme 16:**
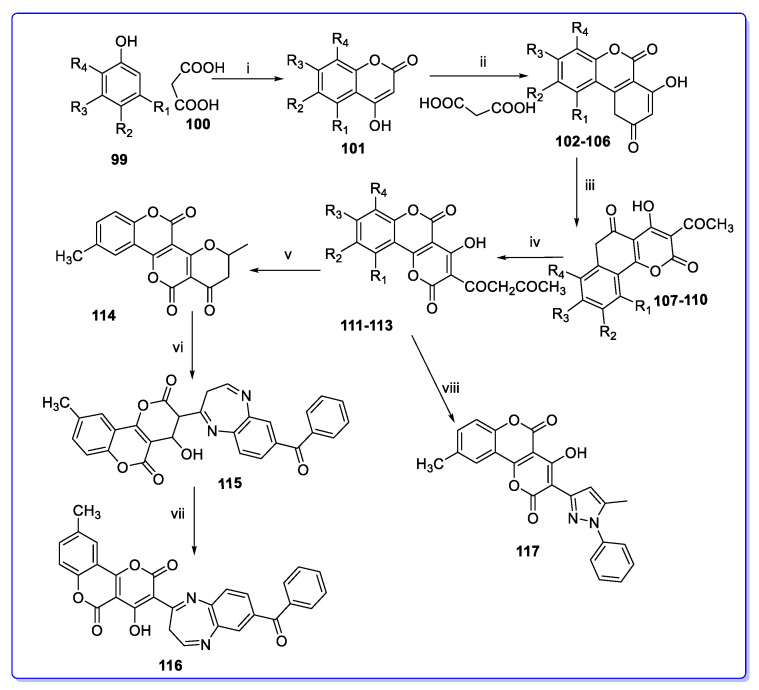
Synthetic pathways for the synthesis of hybrids **116** and **117**. Reagents and conditions: (**i**) POCl_3_, ZnCl_2_; (**ii**) POCl_3_, ZnCl_2_ (**iii**) gla CH_3_COOH, POCl_3_ (**iv**) ethylacetate, Na; (**v**) Ethanol, HCl (**vi**) 3,4-DABP (**vii**) POCl_3_, 1,2-dichloromethane, Pd-C/H_2_ (**viii**) Ph-NHNH_2_.

The reaction of malonic acid and phenol in the presence of phosphorus oxychloride and zinc chloride afforded the 4-hydroxycoumarin derivatives. The further treatment of these 4-hydroxycoumarin derivatives with zinc chloride, malonic acid and phosphorus oxychloride resulted in the production of 4-hydroxy-2-methylenepyrano [3,2-*c*] chromene-2,5-dione (**102**–**106**). Compounds (**102**–**106**) were further acylated to give various acetyl-substituted compounds (**107**–**110**). Compounds (**107**–**110**) were further reacted with sodium metal and ethyl acetate to afford 4-hydroxy-3-(3-oxobutanoyl) pyrano [3,2-*c*] chromene-2,5-dione (**111**–**113**). The acidic hydrolysis of compounds (**111**–**113**) afforded hybrid **114**. Using phenyl hydrazine and 3,4-diaminobenzophenone, compounds (**111**–**113**) underwent cyclization to form 3-(7-benzoyl-3*H*-benzo [b] [1,4] diazepin-2-yl)-4-hydroxypyrano [3,2-*c*] chromene-2,5-dione **115** and **116** and 4-hydroxy-3-(5-methyl-1-phenyl-1*H*-pyrazol-3-yl) pyrano [3,2-*c*] chromene-2,5-dione **117**, respectively, in good yield of 56–82% (Scheme 16).

The molecular docking studies against the RNase H protein active site indicated that compounds **105**, **103**, **104**, **101** and **99** have the best binding affinity against the protein. Important hydrophobic interactions with binding pocket residues were also observed in the active site of the protein. The most interesting derivative was compound **105** for its ability to inhibit both RNase H and HIV-1 IN in the low micromolar range. To maintain excellent potency against PR while obtaining RT inhibition, coumarin moieties were fused into HIV-1 protease inhibitors to produce a more potent hybrid according to “portmanteau inhibitors” or designed multifunctional ligands (DMLs). Zhu and co-workers designed various coumarin hybrids with different linkers that exhibited weak inhibition of RT and excellent potency against PR [54].

**Scheme 17:**
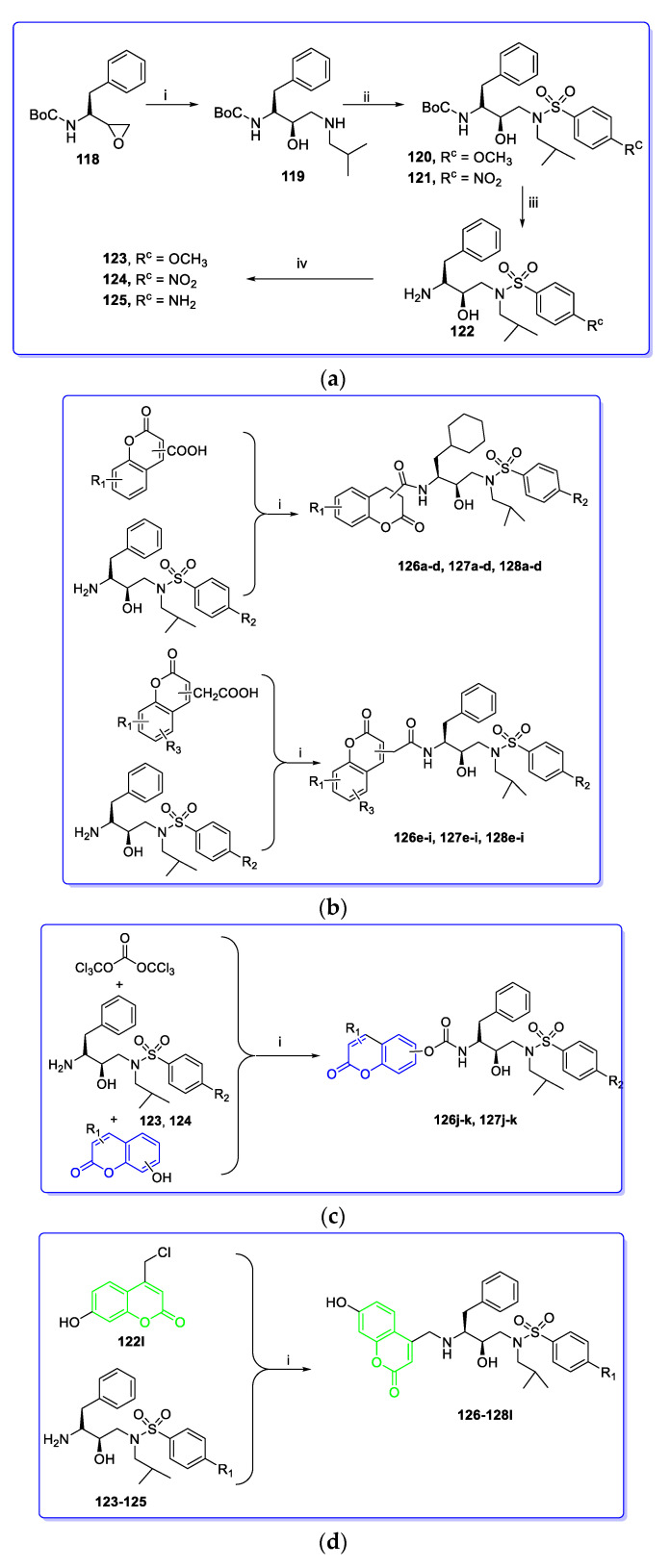

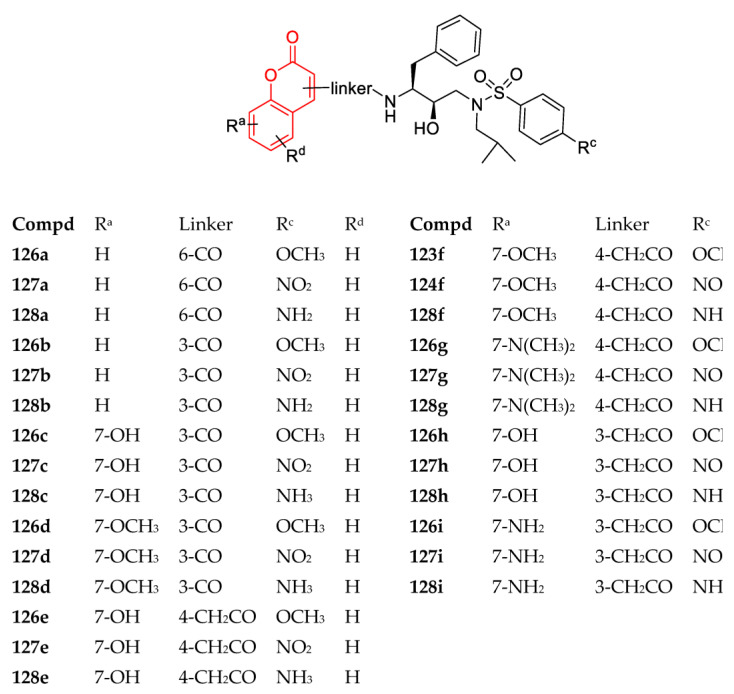
Molecular structure of compounds **126a**–**128i**. (**a**) A synthetic pathway for the synthesis of amine derivatives. Reagents and conditions: (**i**) CH_3_CN, *i*-BuNH_2_, 80 °C, 6 h; (**ii**) DIEA, aryl sulfonyl chloride, DMAP(Cat.), THF, 0 °C~r.t, 3–5 h; (**iii**) CH_2_Cl_2_-CF_3_COOH (1:1), 0 °C~r.t, 3 h; (**iv**) 50 psi, H_2_ (gas), 10% Pd/C, CH_3_OH, r.t, 2 h. (**b**) Synthetic pathways for the synthesis of coumarin–amide hybrids **126a**–**128i**. Reagents and conditions: (**i**) HOBt, EDCI, anhydrous DMF, DMAP, Argon, 0 °C~r.t, 3 h. (**c**) Synthetic pathways for the synthesis of coumarin–carbide hybrids **126j**–**128k**. Reagents and condition: (**i**) Anhydrous DCM, DIEA, anhydrous THF, 0 °C~r.t, 1.5 h. (**d**) Synthetic pathways for the synthesis of coumarin–amine hybrids **123**–**125l**. Reagents and conditions: (**i**) anhydrous EtOH, DIEA, reflux, 7 h.

The synthetic procedures began with the production of amine derivatives (**122**–**125**) which were afforded from commercially available (2*S*, 3*S*)1,2-epoxy-3-(boc-amino)-4-phenylbutane as shown in Scheme 17a. Scheme 17b depicts the synthetic pathways for synthesising coumarin–amide hybrids **123a**–**125i**, which proceed from the coupling of amines **123**–**125** with coumarin acids **122a**–**d** under an EDCI/HOBt/DMAP-mediated coupling method. The synthesis of coumarin–carbamate hybrids **123j**–**124k** shown in Scheme 17c shows the reaction of amines **123**, **124** with hydroxycoumarins **122j**, **122k** using bis(trichloromethyl) carbonate (BTC) as a condensing agent adapting to a one-pot reaction. The refluxing of chlorocoumarin **122** and amines **123**–**125** using DIEA as a catalyst afforded the target coumarin–amine hybrids **123**–**125l** with a wide variation in the yield ranging from 22–98%, as shown in Scheme 17d.

RT activity and the HIV-1 PR assay were used to test all the synthesised hybrids. Coumarin–amide hybrids show PR inhibition with an IC_50_ value range of (298.4 nM–0.40 nM) which indicates that the derivatives are active PR inhibitors except for hybrids **127h** and **127i** (557 nM and 563 nM, respectively). A four-fold activity with an IC_50_ value of 0.40 nM was observed in hybrid **128a**, indicating the best activity. A comparable potency as darunavir (1.72 ± 0.73 nm) was noticed in hybrid **126e** with an IC_50_ value of 1.62 nM, and a 54.46% inhibition ratio against WT HIV-1 was also noticed at a concentration of 100 nM. Generally, the synthesised hybrids show better inhibitory activity against PR than RT inhibition. Hybrid **128b** with an IC_50_ value of 75.25 µM against RT revealed its weakness when compared to the potency of efavirenz with IC_50_ of 0.091 ± 0.008 µM.

## 4. Coumarin Hybrids with Weak Activity against HIV Infections

**Scheme 18:**
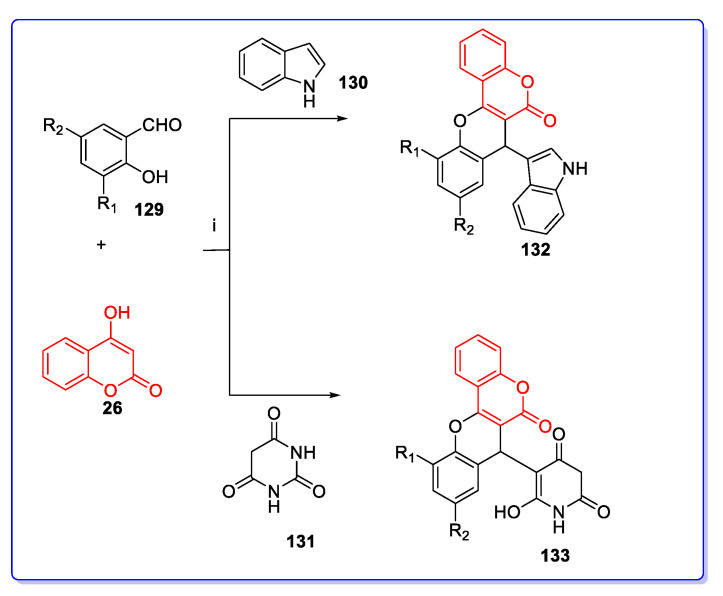
Synthesis of novel chromeno–-chromenone hybrids. Reagents and conditions: (**i**) EtOH, *L*-proline, reflux, 12–14 h, 78–90%.

Through the synthesis of modified aminocoumarin as a leaving group, 7-amino-4-carbamoylmethylcoumarin (ACC) was synthesised via the solid-phase synthetic procedure for the inhibition of HIV-PR. However, relatively weak activity was observed in all the synthesised hybrids [55]. 6,6,10,10-Tetramethyl-6*H*,10*H*-dipyranocoumarin (dipetalactone) also exhibited no anti-HIV activity (using HIV-1 strain IIIB of human immunodeficiency virus type 1). The molecular docking studies revealed weak to no significant amino acid interactions occurring within the binding pocket of the HIV proteins, which demonstrated its weak activity [56]. Drzewiecka et al. suggested that if only a methyl group is substituted in the dipyranocoumarin system, the compound will have no biological activity [56]. Novel chromeno–chromenones exhibit weak anti-HIV activity and were synthesised by the reaction of indole catalysed by *L*-pyroline and 4-hydroxycoumarin [57]. The synthetic pathway (Scheme 18) depicts the reaction between the coumarin derivative and the indole. Some derivatives of coumarins have been synthesised and have exhibited weak to no activity against HIV [58,59].

## 5. POM Analyses: Identification of Anti-HIV Pharmacophore Sites

The first point of the conclusion is that comparing the activities of molecules as cited above by calculating chemical parameters and using theoretical calculations has now become much easier. Developing technology and breakthroughs have improved both programmes and computers. The use of DFT theory and docking analysis is a robust tool that provides valuable information on the chemical, electronic, and physical properties of molecules, allowing us to explain their biological activities; however, only several of these techniques are effective in some situations. This is especially true when the prominent, active one is a metabolite, not the parent molecule. Hence, the theoretical study of prodrugs is a mistake, and this should be taken into consideration as well as stopped. As an alternative solution, we developed the POM (Petra/Osiris/Molinspiration) theory in collaboration with the NCI and TAACF of the USA [60,61,62,63,64].

**Figure 11:**
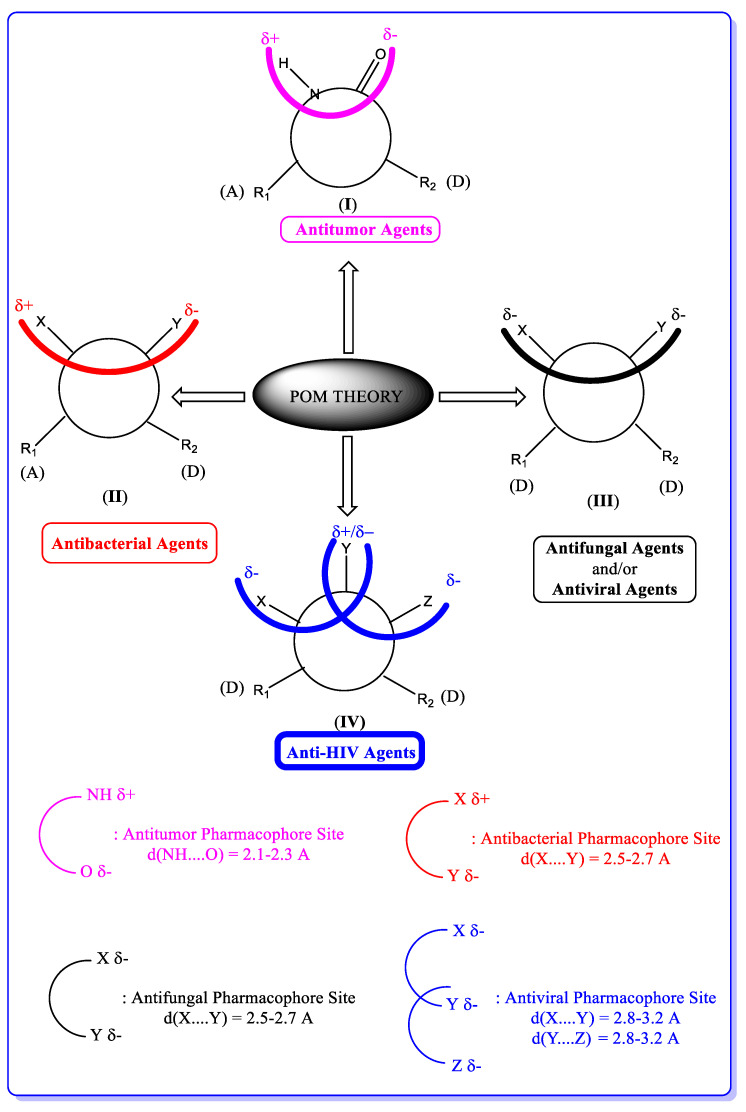
The Concept and Applications of POM Theory in the identification and optimisation of pharmacophore sites of various classes of drugs developed by Prof. T. Ben Hadda (the principal inventor of POM Theory) in collaboration with the NCI and TAACF of the USA [76,77].

The POM theory, invented by the group of Taibi Ben Hadda in collaboration with the American National Cancer Institute (NCI) and Tunisian–American Association for Cancer Research and Training Foundation (TAACF), led us to real success in the pharmacology and drug design fields [65,66,67,68,69,70,71,72]. Here we treat the coumarin moiety of the selected compounds to clarify the origin of their antiviral activity and to identify their pharmacophore sites according to the POM organigram (Figure 11).

Current research has been encouraged by the potential pharmacological properties of coumarin compounds. As an extension of our study, it has also focused more on the identification of novel antiviral heterocyclic compounds for therapeutic purposes [73]. The objective of this research is to evaluate a series of hybrid coumarin congeners for their activities against HIV infections. Moreover, POM analyses were conducted to explain the experimental results of biological activity [65,69,70,71,72,74,75].

**Figure 12:**
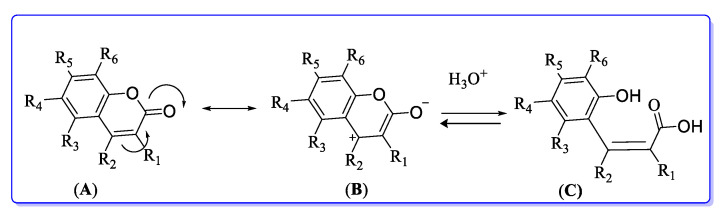
Opening/closing ring of coumarin moiety [75].

The identification of the type of pharmacophore sites of these compounds was derived from the physical and chemical properties of the metabolites of the tested coumarin-hybrid by using the bioinformatics POM platform. Although the mechanism of the ring opening of coumarin was not well clarified, it appeared that the first step essentially seems to be the formation of the enolate from (A), resulting in the formation of the hemiacetal (B). The hemiacetal, being very unstable, opens immediately to a new formation (C) (Figure 12).

**Figure 13:**
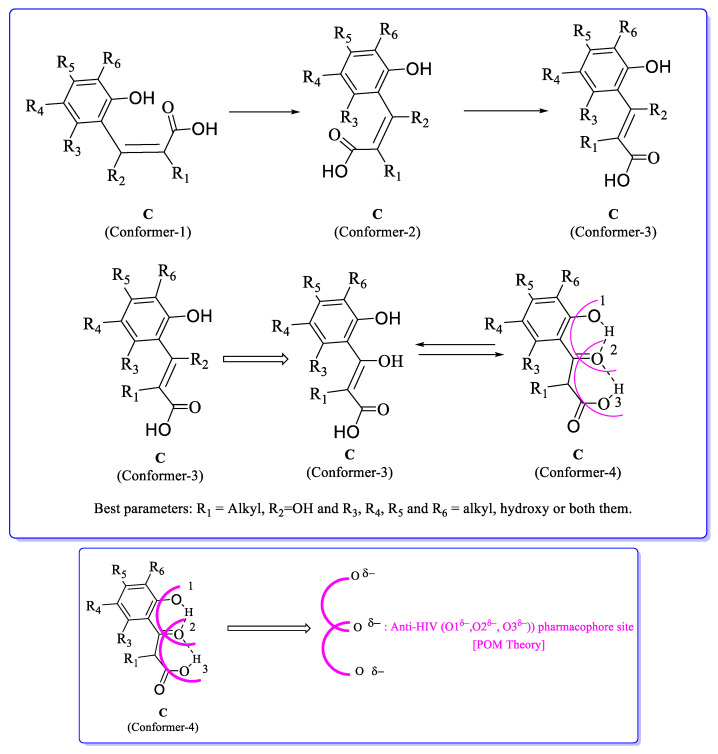
Identification of antiviral pharmacophore sites of coumarin-hybrid compounds.

The probable mechanism of the opening/closing ring reaction was described previously after 1980 [75]. Unfortunately, no chemist or pharmacologist, until now, has indicated the impact and importance of these processes on the bioactivity of coumarin. Therefore, it was for the first time that we attempted to do it, and hopefully it will be of benefit in the future. In contrast to all substituents (R_1_, R_3_, R_4_, R_5_ and R_6_), the sole and exceptional substituent is R_1_, which plays a crucial role in the arrangement of the antiviral pharmacophore site according to POM theory (Figure 11 and Figure 13). Once again, POM theory works, gives more clarification, and helps in the drug design of new coumarin-hybrid candidates, allowing for more efficiency and selectivity.

## 6. Conclusions and Future Viewpoint

Although it still has its own drawbacks and difficulties, the introduction of the HAART regimen has changed the dynamics of HIV disease, converting it from a nearly fatal illness into a chronic but seemingly stable condition. These setbacks among other reasons, made medicinal chemists and scientists plunge headfirst into the field of drug discovery due to the obvious necessity of developing a novel anti-HIV drug candidate having less prevalent complications than the HAART regimen. HAART has reduced the deadly impact of AIDS/HIV from its original status as a deadly disease to what is now considered a manageable infection and consequently scaled down the far-reaching destruction of the ailment throughout the world. Unfortunately, some patients suffer various side effects as well as the development of multi-drug resistance, thus inhibiting a smooth treatment for patients.

Natural sources, which are generally applicable to various infections, are currently one of the most common sources of medication. This led to the exploration of the coumarin moiety due to its versatility and druggability, as seen in the development of warfarin (anti-coagulants), methoxsalen (anti-dermatosis), novobiocin (antibiotics), etc. (+)-Calanolide A, a clinically-evaluated coumarin derivative, inhibits RT, IN, PR, Tat, and Vpr. The review considered in great detail the many reactions and pathways used in synthesis that resulted in the development of numerous coumarin hybrids with anti-HIV activity.

This compilation has shown that coumarin hybrids possess multiple anti-HIV mechanisms or can mitigate side effects, making their hybridization an effective strategy for developing novel agents with high potency against drug-resistant HIV strains and low toxicity compared to that experienced by patients under the current regime of HAART. Several potent hybrids have been designed and synthesised using coumarin synthesis methods. They can reduce toxicity and atone for the hybrid’s negative effects when combined with anti-HIV actions of coumarin hybrids. The structure–activity relationship of the synthesised hybrids will assist in understanding the correlation between their structural properties and anti-HIV activity, enabling the development of more effective anti-HIV hybrids.

## Data Availability

The data and materials supporting the findings or analyses in their study are all readily accessible upon request.

## Abbreviations

POM	Petra/Osiris/Molinspiration
SAR	Structure–Activity Relationship
HIV	Human Immunodeficiency Syndrome
AIDS	Acquired Immunodeficiency Syndrome
HAART	Highly Active Antiretroviral Therapy
RT	Reverse Transcriptase
WHO	World Health Organisation
WOS	Web of Science
ART	Antiretroviral therapy
cART	Combined Antiretroviral Therapy
IN	Integrase
DNA	Deoxyribonucleic acid
VPR	Viral Protein Regulator
DCK	Hydroxymethyl (3′*R*,4′*R*)-3′,4′-di-O-(*S*)-camphanoyl-(+)-cis-khellactone
AZT	Azidothymidine
PETT	Phenyl Ethyl Thiazolyl Thiourea
MTT	4,5-dimethyl thiazol-2-yl)-2,5-diphenyl tetrazolium bromide
TEA	Triethylamine
DMF	Dimethyl formamide
EFV	Efavirenz
DABCO	1,4-diazabicyclo[2.2.2]octane/triethylenediamine
PR	Protease
DMLs	Designed Multifunctional Ligands
EDCI	1-Ethyl-3-(3-dimethylaminopropyl)carbodiimide
DMAP	4-Dimethylaminopyridine
BTC	Bis(trichloromethyl)carbonate
DIEA	N,N-Diisopropylethylamine
DCM	Dimethylamonopyridine
DCC	Dicyclocarbodiimide
WT	Wild-Type
DFT	Density Functional Theory
NCI	American National Cancer Institute
TAAF	Tunisian–American Association for Cancer Research and Training Foundation
